# Effects of Dietary Protein and Energy Levels on Growth Performance, Nutrient Digestibility, and Serum Biochemical Parameters of Growing Male Mink (*Neovison vison*)

**DOI:** 10.3389/fvets.2022.961461

**Published:** 2022-07-18

**Authors:** Feifei Han, Jing Wang, Lihong Chen, Wei Zhong

**Affiliations:** ^1^Animal Science and Technology College, Jilin Agricultural Science and Technology College, Jilin, China; ^2^State Key Laboratory of Special Economic Animal Molecular Biology, Institute of Economic Animal and Plant Science, Chinese Academy of Agriculture Sciences, Changchun, China

**Keywords:** mink, protein, energy, growth performance, nutrient digestibility, blood character

## Abstract

The objective of this experiment was to determine the optimum dietary metabolic energy (ME) and crude protein (CP) levels of growing male mink. One hundred forty-four healthy male minks at 75 days were randomly allocated into the six groups with 24 replicates, which was one mink for each replicate. The mink were fed six experimental diets with two CP levels (31.59 and 35.63%) and three ME levels (14.17, 15.96, and 17.73 MJ/kg) for a 7-day preliminary period and then for an 88-day experimental period. The final body weight (BW), average daily gain (ADG), feed conversion ratio (FCR), fat digestibility, energy intake, the concentration of glucose (GLU), and low-density lipoprotein (LDL) of the mink were significantly increased by the CP or ME levels (*P* < 0.05). In addition, CP levels significantly (*P* < 0.01) increased the N intake and N retention. Dietary ME levels increased the utilization of gross energy. Obviously, there were significant CP × ME interactions for the final BW, ADG, fat digestibility, energy utilization, GLU, LDL (*P* < 0.01), and triglyceride contents (*P* < 0.05). Therefore, the optimum CP and ME levels were 35.97% and 18.18 MJ/kg, which can improve growth, enhance nutrient digestion, and promote blood lipid metabolism in growing mink.

## Introduction

Mink (*Mustela vison*) are fur-bearing animals endemic to North America and have been introduced into China in the 1980s (1, 2). Mink are present in China as domestic populations harbored under farm conditions (3). At the same time, as strict carnivorous animals, mink consume a diet containing mainly protein and fat of animal origin (4, 5). In general, mink demand higher dietary protein and energy levels than those other domestic animals (6). Proper dietary protein and energy are necessary for the growth and productivity of mink (7, 8) and excessive or insufficient ingestion results in corresponding clinical signs. Research showed that for mink, the optimum dietary protein levels in the growth period were 42% metabolic energy (ME) and the digestive protein requirement in the growth period is 30% ME for mink (9). When the dietary protein is 32% and the fat level is 20–30%, the nutrient utilization rates are higher and during the growth period, male mink can achieve better growth performance. Healthy male mink were fed six experimental diets with two protein levels (32 and 36%) and three fat levels (10, 20, and 30%); when the dietary fat level was 10%, the growth rate was significantly (*P* < 0.01) lower than that of the other groups and mink health can be affected (10). In addition, the maintain ME requirement in the mink growth period is 607–680 KJ/kg BW^0.75^ (11, 12). Moreover, protein metabolism has a strong connection with energy metabolism (13–15). Activating natural energy metabolism can improve protein synthesis (16) and an increase in dietary ME intake of 1 MJ/day decreased dietary lysine disappearance by 0.74 g/day (17).

Although some results related to protein or energy metabolism in mink can be found in the literature, research on the effects of dietary protein and energy levels on growing mink is lacking. Moreover, due to the differences in mink species, feeding conditions, and nutritional values of the feed ingredients in different regions, there is no consensus on the energy requirements of mink (18, 19). Therefore, this trial aimed to investigate the effects of dietary crude protein (CP) and ME levels on growth performance, nutrient digestibility, nitrogen and energy balance, and physiological blood parameters in growing male mink, to evaluate the optimal CP and ME levels in the diet of growing mink, and provide a reference for improving the standard nutrition requirements for mink in China.

## Materials and Methods

### Ethics Approval and Consent to Participate

All the animals used in the study were treated following the guidelines established by the Council of China Animal Welfare. Protocols of the experiments were approved by the Animal Ethics Committee of the Chinese Academy of Agricultural Sciences (CAAS).

### Experimental Design, Diet, and Management

All the mink in this study were fed in Changbai Mountain Wildlife Resources Key Field Scientific Observation and Experiment Station. The diet was based on expanded soybean, soybean meal, chicken meal, and fish meal (Tables 1, 2) and was formulated to meet the nutrient requirements of growing mink. The experiment employed a 2 × 3 factorial design, with two CP levels (31.59 and 35.63%) and three ME levels (14.17, 15.96, and 17.73 MJ/kg).

**Table 1:** The experiment designs.

**“C” “P” (%)**	**ME (MJ/Kg)**		
	**14.17**	**15.96**	**17.73**
31.59%	A	B	C
35.63%	D	E	F

**Table 2:** Composition and nutrient level of experiment diet (%; air-dry basal).

**Items**	**Groups**					
	**A**	**B**	**C**	**D**	**E**	**F**
Ingredients, %						
Extruded corns	45	37	29	37.5	30	23
Expanded soybean	2	2	2	4	4.5	2
Soybean meal	3	2	3	2	2	2
Born meat meal	20	20	20	20	20	20
Corn protein meal	2	2	2	4	2	3
Chicken meal	4	7	8	7	7	8
Fish meal	15.5	15	14.5	18	20.5	21
Lysine	0.3	0.3	0.3	0.3	0.3	0.3
Methionine	0.5	0.5	0.5	0.5	0.5	0.5
Salt	0.2	0.2	0.2	0.2	0.2	0.2
Dicalcium phosphate	0.5	0.5	0.5	0.5	0.5	0.5
soybean oil	6	12.5	19	5	11.5	18.5
Premix^a^	1	1	1	1	1	1
Total	100	100	100	100	100	100
Nutrient levels^b^						
Gross energy (MJ/kg)	18.86 (0.05)	21.20 (0.13)	22.42 (0.15)	19.55 (0.19)	20.61 (0.01)	22.07 (0.01)
Metabolic energy (MJ/kg)^c^	14.05	16.61	17.52	14.91	16.21	18.18
Crude protein, % DM	31.17 (0.05)	31.50 (0.65)	32.09 (0.04)	35.78 (0.06)	35.14 (0.06)	35.97 (0.08)
Ether extract, % DM	12.07 (0.05)	18.83 (0.06)	25.31 (0.04)	12.77 (0.05)	17.60 (0.04)	23.54 (0.07)
Carbohydrate, % DM	43.27	36.17	29.40	36.89	31.86	26.49
Calcium, % DM	3.03 (0.13)	3.21 (0.04)	3.16 (0.09)	2.93 (0.13)	2.71 (0.12)	3.18 (0.03)
Total Phosphorus, % DM	1.33 (0.02)	1.34 (0.03)	1.33 (0.03)	1.47 (0.02)	1.52 (0.01)	1.53 (0.02)
Lys, % DM	1.85	1.78	1.76	1.99	2.09	2.05
Met, % DM	1.06	1.03	1.01	1.12	1.13	1.13
Cys, % DM	0.31	0.28	0.27	0.33	0.31	0.30
Metabolic energy as fat, % ME	23.18	36.95	49.63	25.32	36.63	47.59
Metabolic energy as protein, % ME	32.79	27.97	27.24	36.64	32.63	29.93
Metabolic energy as carbohydrate, % of ME	42.27	30.17	22.91	34.68	28.00	21.34

*^a^One kilogram of premix contained the following: Vitamin A, 625,000 IU; Vitamin D_3_, 200,000 IU; Vitamin E, 18,500 mg; Vitamin K_3_, 200 mg; Vitamin B_1_, 1,250 mg; Vitamin B_2_, 900 mg; Vitamin B_6_,750 mg; Vitamin B_12_, 2.25 mg; Vitamin C, 26,000 mg; biotin, 10 mg; folic acid, 50 mg; nicotinic, 2,500 mg; pantothenic acid, 1,750 mg; choline, 120,000 mg; Fe, 9,600 mg; Cu, 1,200 mg; Zn, 9,600 mg; Mn, 4,800 mg; I, 120 mg; Se, 30 mg; Co, 48 mg*.

*^b^Gross energy, Crude protein, Ether extract, Calcium, Total Phosphorus were measured values (mean ± SEM; n = 3), While the others were calculated values*.

*^c^ME was calculated by follows: ME (MJ/Kg) = GE (MJ/Kg) × MEI/GEI (%)*.

One hundred and forty-four 9-week-old male black mink were randomly distributed into the six treatment groups with similar weights (1206.06 ± 91.17 g), 24 replicates per group and one mink per replicate. Animals were individually housed in standard cages (100 × 70 × 60 cm). Mink were fed two equal-sized meals daily at 07:30 and 15:30 h and collected the leftover before each feeding. Mink were given free access to water. Mink were fed for a 7-day adaptation period and for an 88-day trial period from 2 July to 29 September.

### Evaluation of Growth Performance

The fasting live weight of mink on an empty stomach on the first day of the formal period was the initial weight and the final weight was determined after the end of the experiment. The body weights of the mink were measured and recorded in the morning before feeding every 15 days. Feed intake was recorded daily during the whole experimental period and the average daily gain (ADG) and feed conversion ratio (FCR) were calculated for each mink individually.

### Digestibility Trial

On day 50 of the growth period, eight animals from each treatment group were selected randomly and housed individually in metabolic cages that allowed the separation of urine and feces for 3 days of digestive, nitrogen, and energy balance trials to determine nutrient digestibility. Feed was sampled for further analysis. The excretions were collected each day. According to the volume of urine, 10 ml of 10% H_2_SO_4_ and five drops of methylbenzene per 100 ml of urine were used to combine the nitrogen. Urine samples were collected in plastic bottles and stored at −20°C until analysis. Fecal and feed samples were dried to a constant weight in a forced air drying oven at 65°C, then ground to pass through a 40-mesh sieve, and stored for further analysis.

### Serum Biochemical Parameters Analysis

At the end of the experiment, eight minks were selected randomly from each group and blood samples were taken *via* the toe clip from the mink in the morning before feeding. Blood samples were collected in two separate tubes with 10 μl procoagulant substances. Samples were quickly transferred to the laboratory where plasma and serum were obtained by centrifuging the tubes for 15 min at 3,500 rpm. The serum was frozen at −80°C for later analysis.

### Chemical Analysis

The chemical compositions of the diets, feces, and urine were analyzed by the Association of Official Analytical Chemists (AOAC) International methods (AOAC, 2005). The dry matter (DM) of the diets and fecal samples was determined by drying the feed or fecal samples at 105°C to constant weight (method 920.39) (AOAC, 2005). Nitrogen was measured by FOSS Kjeltec 8400 analyzer (Foss, Hillerød, Denmark) and CP was calculated as *N* × 6.25 (method 984.13) (AOAC, 2005). The ether extract in the feces and feed was determined using the Buchi Extraction System B-811 (method 920.39) (AOAC, 2005). The gross energy content of the diets and feces was measured with an adiabatic bomb calorimeter with benzoic acid used as a standard. The concentration of GE in each urine sample was measured after 8 ml of the sample was dripped onto two pieces of filter paper in a special crucible and dried for 8 h at 65°C in a drying oven and, then, the energy content was determined using an adiabatic bomb calorimeter (IKAC2000, Calorimeter, IKA Company, Germany). Lysine was determined by hydrolyzing samples with 6 mol/l HCl for 24 h at 110°C (AOAC, 2007) and analyzed using an amino acid analyzer (Hitachi L-8900; Hitachi Ltd., Tokyo, Japan). Methionine and cysteine were determined as methionine sulfone and cysteic acid, respectively, after cold performic acid oxidation overnight and hydrolysis with 7.5 mol/l HCl for 24 h at 110°C, using an amino acid analyzer (Hitachi L-8900; Hitachi Ltd., Tokyo, Japan).

Serum total protein (TP), albumin (ALB), globulin (GLOB), urea, alkaline phosphatase (ALP), aspartate aminotransferase (AST), alanine aminotransferase (ALT), glucose (GLU), cholesterol (CHO), triglyceride (TG), low-density lipoprotein (LDL), high-density lipoprotein (HDL), and lactate dehydrogenase (LDH) were determined by an automatic biochemistry analyzer (Vita Lab Selectra-E, Holland) and the test kits noted above were purchased from Nanjing Jiancheng Biochemical Corporation (Nanjing Jiancheng Biochemical Corporation, Nanjing, China).

### Statistical Analysis

The data were initially checked for normality using the PROC UNIVARIATE procedure. Statistical analysis was conducted using the generalized linear model (GLM) procedure of SAS 9.2 (SAS Institute Incorporation, Carr, North Carolina, USA). The data were analyzed as a 2 × 3 (CP × ME) factorial arrangement of treatments by two-way ANOVA with a model that included the main effects of CP and ME and their interaction. The means were compared by Duncan's multiple comparison tests to determine whether there were significant effects between them. All the data were expressed as the means ± SEM. *P* < 0.05 was considered significantly different among the means, *P* < 0.01 was considered extremely significant difference among the means, and *P* > 0.05 was considered no significant difference among the means.

## Results

### Growth Performance

As shown in Table 3, mink fed the 35.63% CP diet had a higher final BW, ADG, average daily feed intake (ADFI), and FCR than those fed the 31.59% CP diet (*P* < 0.01). The final BW, ADG, and FCR of the mink in the 17.73 MJ/kg ME group significantly increased than those in the other groups (*P* < 0.01). ADFI was not affected by dietary ME levels (*P* > 0.05). There was a significant interaction between ME and CP in the final BW, ADG, ADFI, and FCR (*P* < 0.01). The final BW and ADG of the mink in the F diet group were higher than those in the A and B diet groups (*P* < 0.01) and the ADFI was higher in the F diet group than those in the A, B, and C diet groups (*P* < 0.01). The FCR of mink was higher in the C diet group (*P* < 0.01) than in the other groups. Mink fed the diets of the F group had better growth performance than the other groups.

**Table 3:** Effects of energy and protein levels on growth performance of mink during growing period^1^.

**Items**		**Initial weight (g)**	**Final weight (g)**	**ADG (g)**	**ADFI (g)^**2**^**	**FCR**
Groups	A	1190.00	1493.57^Cb^	3.45^Bb^	97.96^CDc^	30.55^Aa^
	B	1222.31	1533.08^BCb^	3.53^Bb^	92.12^Dc^	27.92^ABa^
	C	1194.29	1807.33^ABa^	6.97^Aa^	104.33^BCDbc^	14.40^Cb^
	D	1214.67	1750.67^ABCa^	6.09^ABa^	114.62^ABCabc^	19.64^BCb^
	E	1229.09	1770.91^ABCa^	6.16^ABa^	119.71^ABab^	19.18^BCb^
	F	1194.31	1934.38^Aa^	8.41^Aa^	128.04^Aa^	17.94^BCb^
MSE		9.95	32.82	0.36	2.76	1.31
Main effects
Dietary CP level/%	31.59%	1201.43	1617.86^B^	4.73^B^	98.44^B^	23.52
	35.63%	1210.69	1825.95^A^	6.99^A^	120.55^A^	18.94
Dietary ME level /(MJ/Kg)	14.17	1202.76	1626.55^B^	4.82^B^	107.48	24.31^A^
	15.96	1225.42	1642.08^B^	4.84^B^	104.85	23.89^A^
	17.73	1194.16	1872.90^A^	7.71^A^	115.40	16.05^B^
*P*-Value	Dietary CP level	0.1691	0.0016	0.0024	<0.0001	0.0572
	Dietary ME level	0.8838	0.0018	0.0014	0.2156	0.0064
	Interaction	0.8286	0.0001	<0.0001	0.0001	0.0004

*In the same item and column, values with different lowercase superscripts mean P < 0.05, and with different capital letter superscripts mean P < 0.01, while with same or no letter superscripts mean no significant difference (P > 0.05).The same as below*.

*^1^Data are expressed as least squares means with pooled SEM; n = 24 per treatment*.

*^2^ADFI, average daily feed intake*.

### Nutrients Digestibility

The DM digestibility and fat digestibility of the mink fed the 35.63% CP diet significantly were higher (*P* < 0.01) than those of the mink fed the 31.59% CP diet (Table 4; *P* < 0.01 or *P* < 0.05). CP levels had no effect on protein digestibility (*P* > 0.05). ME levels had no effect on DM and protein digestibility (*P* > 0.05). Fat digestibility increased as ME levels increased in the diet (*P* < 0.01) and maximum digestibility was observed in the 17.73 MJ/kg group. There was no significant interaction observed between the ME and CP levels in terms of the DM and protein digestibility (*P* > 0.05), but there was a significant interaction in fat digestibility (*P* < 0.01). Mink fed the diets of the F group had higher fat digestibility and DM digestibility compared to the other groups.

**Table 4:** Effects of energy and protein levels on nutrient utilization of mink during growing period^1^ %.

**Items**		**DM digestibility**	**Protein digestibility**	**Fat digestibility**
Groups	A	69.52	78.59	67.81^Cd^
	B	72.45	78.43	81.90^ABbc^
	C	72.85	79.03	86.27^Aab^
	D	73.55	81.15	74.23^Bc^
	E	73.92	80.03	84.76^ABab^
	F	76.38	80.45	92.36^Aa^
SEM		0.62	0.54	1.86
Main effects
Dietary CP level/%	31.59%	71.76^B^	78.70	78.21^b^
	35.63%	74.60^A^	80.60	83.20^a^
Dietary ME level/(MJ/Kg)	14.17	71.82	80.05	69.34^Bc^
	15.96	73.13	79.17	83.20^Ab^
	17.73	74.50	79.69	89.08^Aa^
*P*-Value	Dietary CP level	0.0091	0.0540	0.0407
	Dietary ME level	0.0849	0.6482	<0.0001
	Interaction	0.0616	0.6426	<0.0001

*^1^Data are expressed as least squares means with pooled SEM; n = 8 per treatment. The same as below*.

### Nitrogen Balance

Nitrogen (N) intake, fecal N, urinary N, and N retention of the mink fed the 35.63% CP diet were higher than those fed the 31.59% CP diet (Table 5; *P* < 0.01). However, dietary CP levels had no effects on the net protein utilization (NPU) and biological value (BV) of the protein in the mink (*P* > 0.05). ME levels had no significant effects on N intake, fecal N, urinary N, N retention, NPU, and BV (*P* > 0.05). There was significant interaction observed between the ME and CP levels in terms of N intake and N retention (*P* < 0.01), as well as the fecal N and urinary N (*P* < 0.05). However, there was no significant CP × ME interaction for NPU and BV of the protein in the mink (*P* > 0.05), whereas the NPU and BV in the F diet group were higher than those in the other groups. The N intake, N retention, and fecal N of the mink in the F diet group were significantly higher than those in the A, B, and C diet groups (*P* < 0.01 or *P* < 0.05). The nitrogen drains in fecal N of mink in the F diet group were higher than those in the A, B, and C diet groups (*P* < 0.05), whereas no significant difference was found among the D, E, and F diet groups (*P* > 0.05). Urinary N was significantly higher in the F diet group than in the B diet group (*P* < 0.05), whereas no significant difference was found among the other groups (*P* > 0.05).

**Table 5:** Effects of energy and protein levels on nitrogen balance of mink during growing period.

**Items**		**Intake N (g/d)**	**Fecal N(g/d)**	**Urinary N(g/d)**	**N Retention (g/d)**	**NPU (%)**	**BV of protein (%)**
Groups	A	4.89^B^	1.04^bc^	2.15^a^	1.70^Bd^	34.80	44.03
	B	4.64^B^	1.00^c^	1.77^b^	1.87^Bcd^	39.49	52.00
	C	5.36^B^	1.13^bc^	2.20^a^	2.03^Bbcd^	38.35	48.21
	D	6.56^A^	1.24^abc^	2.68^a^	2.64^ABb^	40.03	49.56
	E	6.73^A^	1.33^ab^	2.88^a^	2.52^ABbc^	37.00	46.10
	F	7.37^A^	1.45^a^	2.51^a^	3.42^Aa^	46.80	57.90
SEM		0.19	0.04	0.11	0.13	1.50	1.79
Main effects
Dietary CP level (%)	31.59%	4.98^B^	1.06^B^	2.04^B^	1.88^B^	37.72	48.28
	35.63%	6.88^A^	1.33^A^	2.68^A^	2.87^A^	41.42	51.35
Dietary ME level (MJ/Kg)	14.17	5.84	1.15	2.45	2.24	37.79	47.19
	15.96	5.61	1.15	2.28	2.17	38.34	49.28
	17.73	6.30	1.28	2.35	2.67	42.29	52.73
*P*-Value	Dietary CP level	<0.0001	0.0015	0.0075	0.0001	0.2594	0.4439
	Dietary ME level	0.1068	0.2773	0.8881	0.1102	0.3802	0.4341
	Interaction	<0.0001	0.0212	0.0423	0.0001	0.3266	0.2309

### Energy Balance

Gross energy intake (GEI), digestible energy intake (DEI), and metabolizable energy intake (MEI) of the mink fed the 35.63% CP diet were significantly higher than those fed the 31.59% CP diet (Table 6; *P* < 0.01), but fecal energy (FE), urinary energy (UE), DEI/GEI, MEI/GEI, and MEI/DEI were not affected (*P* > 0.05). GEI, DEI, and MEI of the mink fed the 17.73 MJ/kg diet were significantly higher than those fed the 15.96 and 14.17 MJ/kg diet (*P* < 0.01), whereas DEI/GEI and MEI/GEI of the mink fed the 17.73 MJ/kg diet were significantly higher than those fed the 14.17 MJ/kg diet (*P* < 0.01or *P* < 0.05). There was significant interaction observed between the CP and ME levels for GEI, DEI, MEI, DEI/GEI (*P* < 0.01), and MEI/GEI (*P* < 0.05) and the highest energy intake and energy utilization were observed in the F diet group. However, there was no CP × ME interaction for FE, UE, and MEI/DEI in mink (*P* > 0.05).

**Table 6:** Effects of energy and protein levels on energy balance of mink during growing period.

**Items**		**GEI KJ/d**	**Fecal energy KJ/d**	**Urinary energy KJ/d**	**DEI KJ/d**	**MEI KJ/d**	**DEI/GEI %**	**MEI/GEI %**	**MEI/DEI %**
Groups	A	1874.67^Dd^	420.01	62.46	1427.66^Dd^	1365.20^Dd^	77.57^Bc^	74.32^b^	95.75
	B	1952.37^CDbcd^	356.14	63.72	1596.23^CDcd^	1532.51^CDcd^	81.65^ABab^	78.37^ab^	95.97
	C	2338.67^BCb^	425.07	89.41	1912.99^BCb^	1823.58^BCb^	81.95^ABab^	78.12^ab^	95.30
	D	2240.37^BCDc^	444.07	83.93	1796.31^BCbc^	1712.38^BCbc^	80.05^ABb^	76.25^b^	95.24
	E	2467.23^ABb^	423.57	103.05	2043.66^Bb^	1940.61^Bb^	82.78^ABab^	78.66^ab^	95.03
	F	2825.88^Aa^	415.08	88.79	2410.81^Aa^	2322.01^ABa^	85.50^Aa^	82.41^a^	96.36
SEM		64.45	14.42	5.63	58.71	56.60	0.62	0.70	0.27
Main effects
Dietary CP level/%	31.59%	2069.62^B^	400.88	73.15	1668.74^B^	1595.59^B^	80.60	77.12	95.65
	35.63%	2500.36^A^	428.55	91.01	2071.81^A^	1980.80^A^	82.65	78.99	95.55
Dietary GE level /(MJ/Kg)	14.17	2072.07^B^	433.76	74.73	1638.32^B^	1563.59^B^	78.99^Bb^	75.42^b^	95.46
	15.96	2190.00^B^	387.26	81.87	1802.74^B^	1720.87^B^	82.18^ABa^	78.51^ab^	94.54
	17.73	2566.04^A^	420.73	89.12	2145.30^A^	2056.18^A^	83.61^Aa^	80.12^a^	95.79
*P*-Value	Dietary CP level	0.0002	0.5356	0.0918	<0.0001	<0.0001	0.0599	0.0699	0.8361
	Dietary ME level	0.0003	0.4963	0.5160	<0.0001	<0.0001	0.0029	0.0109	0.9192
	interaction	<0.0001	0.3803	0.2647	<0.0001	<0.0001	0.0058	0.0236	0.7478

### Serum Biochemical Parameters

The serum GLU and LDL-C of the mink fed the 35.63% CP diet were significantly higher than those of the mink fed the 31.59% CP diet (Table 7; *P* < 0.05), but the CP levels had no effects on the serum TP, Alb, Urea, CHO, TG, and HDL-C (*P* > 0.05). The serum GLU of the mink fed the 17.73 MJ/kg diet was significantly higher than those of the mink fed the 15.96 and 14.17 MJ/kg diet (*P* < 0.01), whereas the LDL-C diet of the mink fed the 14.17 MJ/kg diet was significantly lower than those of the mink fed the 17.73 and 15.96 MJ/kg diet (*P* < 0.05). ME levels had no significant effects on the other indexes (*P* > 0.05). There was significant interaction observed between the CP and ME levels for the serum GLU, LDL (*P* < 0.01), and TG (*P* < 0.05) and the maximum value was observed in the F diet group. There were no CP × ME interactions for any of the serum TP, Alb, Urea, CHO, and HDL-C (*P* > 0.05), but these interactions were higher in the mink fed the F diet group than those in the other diet groups.

**Table 7:** Effects of energy and protein levels on Serum biochemical parameters of minks during growing season.

**Items**		**TP g/L**	**Alb g/L**	**Urea Mmol/L**	**GLU Mmol/L**	**CHO Mmol/L**	**TG Mmol/L**	**LDL Mmol/L**	**HDL Mmol/L**
Groups	A	57.45	21.97	8.13	4.84^B^	6.15	1.27^b^	0.62^Cb^	3.67
	B	56.17	18.38	7.06	4.47^B^	6.22	1.51^ab^	0.66^BCbc^	3.56
	C	56.23	21.46	7.26	4.96^B^	6.34	1.41^b^	0.82^ABCab^	3.75
	D	59.51	21.28	9.09	4.38^B^	6.20	1.44^b^	0.67^BCb^	3.91
	E	57.03	21.33	7.89	5.21^B^	6.94	1.39^b^	0.96^ABa^	3.94
	F	62.11	23.92	8.44	7.18^A^	7.44	1.71^a^	1.00^Aa^	4.28
SEM		0.94	0.48	0.30	0.20	0.17	0.04	0.04	0.11
Main effects
Dietary CP level/%	31.59%	56.66	20.66	7.51	4.76^b^	6.23	1.39	0.69^b^	3.66
	35.63%	59.55	22.18	8.47	5.59^a^	6.86	1.52	0.88^a^	4.04
Dietary ME level /(MJ/Kg)	14.17	58.04	21.76	8.58	4.62^B^	6.18	1.33	0.63^b^	3.80
	15.96	56.63	19.96	7.50	4.86^B^	6.61	1.45	0.82^a^	3.76
	17.73	59.37	22.77	7.89	6.15^A^	6.92	1.57	0.92^a^	4.03
*P*-Value	Dietary CP level	0.1816	0.1093	0.1914	0.0231	0.0929	0.2384	0.0188	0.1069
	Dietary ME level	0.5635	0.0501	0.2166	0.0045	0.3571	0.2160	0.0205	0.6033
	interaction	0.4091	0.1900	0.3988	<0.0001	0.1307	0.0299	0.0029	0.5088

## Discussion

### Growth Performance

In our study, the mink fed the CP 35.63% and ME 17.73 MJ/kg group increased final BW, ADG, ADFI, and FCR, a result that was consistent with previous literature data (18, 20–22). Similarly, it was reported that higher ME levels can improve BW by accumulating body fat reserves (23, 24) and the adipose tissue in mink accounts for approximately a 36% larger proportion of body weight than that of other tissues (25) and improves the FCR in foxes, a carnivorous fur-bearing animal-like mink (26). Our result agreed with the results in the above literature. The ADFI of a mink is usually affected by the palatability and energy levels of its diet (27). The data in this trial show that mink in the 35.63% CP and 17.73 MJ/kg ME groups had the maximum ADFI. This result might occur because the proportion of fish meal in the 35.63% CP diet is higher than in the 31.59% CP diet, which increased feed palatability. Previous research has demonstrated that low protein and energy diets can significantly reduce growth performance and feed conversion efficiency (28). In addition, a low protein and high energy diet or a high protein diet can promote weight gain in mink (9). Our results suggested that the ADG and ADFI of mink in the 31.17% CP and 14.05 MJ/kg diet group and the 31.50% CP and 16.61 MJ/kg diet group were significantly lower than those in the other diet groups, whereas the FCR values in these two groups were significantly higher than those in the other groups. Both the low CP and high ME (CP, 32.09% and ME, 17.52 MJ/kg) and all the high CP (35.63%) diets had beneficial effects on the growth performance of the mink. These data are consistent with the results from the above literature. This consistency is possible because hair and body growth are very dependent on dietary CP levels (29) and the growth rate of mink is relatively fast in the growing season, so the mink have a high protein requirement and respond sensitively to protein deficiency during this period. Research demonstrates that when faced with several different diets varying in protein and energy composition, mink apportioned intake of two foods to maintain a near-constant ratio and amount of protein and energy intake (30). Moreover, energy can partly convert into proteins to meet body requirements, allowing mink to feed on low protein and high energy diets to grow normally. Given the above results, mink can achieve better growth performance when the group CP level is 35.63% and ME level is 17.73 MJ/kg (the actual CP level is 35.97% and ME level is 18.18 MJ/kg in diet).

### Nutrient Digestibility

According to the digestive physiology characteristics, mink are carnivores that prefer to eat high-protein animal feed and have poor utilization of cellulose (31). Both the energy and protein digestibility values tended to increase as the CP or energy level was increased (32). Fat utilization of diets is generally high in fur-bearing animals (12). In this study, the ME levels were regulated by changing the ratio between fat and carbohydrate. Early studies have shown that a major factor influencing the digestibility of nutrients is the fat/carbohydrate (F:C) ratio (26). In our experiment, dietary values of 35.97% CP and 18.18 MJ/kg ME can promote the digestibility of DM and fat of growing mink, which are in agreement with the previous study and a study on pigs (33). Dietary protein or fat increased in the diets and the proportion of plant materials was decreased, which resulted in decreased dietary carbohydrate and cellulose content, thus presumably increasing the digestibility of the nutrients (34).

### Nitrogen Balance

The ADFI and dietary CP levels are the main factors leading to differences in N intake (35, 36). In the present study, the dietary 35.63% CP diet significantly increased N intake, fecal and urinary N excretion, and N retention. Because 35.63% CP group increased ADFI and protein digestibility, N intake and fecal N were increased. The urine N was significantly increased when the mink were fed a high-protein level diet that exceeded the requirement of the animals or dietary amino acid imbalance (7, 37) and this scenario accounted for a large proportion of nitrogen discharge in mink during the growth period (38). Although the CP levels had no significant effects on the NPU and BV in this trial, the NPU and BV values of the mink fed the 35.63% CP diet were higher than those fed the 31.59% CP diet. The data from this trial indicated that the ME levels in the diet did not affect any of the N balance indexes, which is consistent with protein digestibility and protein metabolic parameters in serum in this experiment and other studies (39). When dietary protein can meet the requirements of a mink, its body will not activate natural energy metabolism to improve protein synthesis (16); thus, energy levels have no impact on N metabolism. Research had noted that the CP and ME levels in a diet had no significant interaction with the N utilization of mink (12). In the present study, there was a significant interaction observed between the ME and CP levels in terms of the N intake, N excretion, and N retention, while no CP × ME interaction was observed for the NPU and BV of the mink, a result that is in agreement with the results from Chwalibog and Thorbek. The mink fed the 35.63% CP and 17.73 MJ/kg diet (actual CP level is 35.97% and ME level is 18.18 MJ/kg in diet) increased their N utilization more than the mink fed other diets.

### Energy Balance

The energy intake increased with the increase in CP levels (40). In our study, compared to that in the high-protein diet, the intake of GE, DE, and ME was less in the low-protein level diet. This result was possible because the diet with low-protein levels had lower ADFI, DM, and fat digestibility. In general, energy metabolism measurements were strongly influenced by energy supply (41). In the present study, although the GEI, DEI, and MEI increased with the increase in the ME levels in the diet, the energy excretion was not affected. The greater energy utilization at 17.73 MJ/kg seemed to be due to the addition of oil contributing to a reduced rate of gastric emptying and a reduced passage rate for digesta, which may have contributed to the decreased fecal energy and increased digestibility of energy in this diet (42). Besides that, we found that the DEI/GEI and MEI/GEI were significantly lower in the mink fed the low CP (31.59%) and low ME (14.17 MJ/kg) diet than in those fed the other diets, which also contributed to the reduced gain and the lowest retention of nitrogen. In comparison to the mink fed the other diets, those fed the 35.97% CP and 18.18 MJ/kg diet achieved a higher gross efficiency of energy utilization of energy in this experiment, which could be explained by the difference in the absorption of nutrients (12).

### Serum Biochemical Parameters

The blood concentration of protein and urea reflected the dietary content of protein and can be assumed to be indicators of the metabolism of amino acid metabolism. A study reported that plasma values of total protein and urea were influenced by the dietary protein level (43, 44) and serum biochemical indicators were normal when CP was at the 30% ME level. Urea is the end product of protein catabolism and accumulates during circulation and the lower content of urea is the higher utilization of N (45). In this study, however, the CP levels did not affect the parameters of the serum protein metabolism of the growing mink. The reason for this contrasting observation is likely due to the CP levels set in this study being lower than those in the above studies. The serum GLU and LDL significantly increased as the dietary CP level increased, but the other parameters of serum lipid metabolism were not affected, which indicated that higher CP levels can catalyze the transfer of saccharides and lipids. The ME levels had no effect on serum protein metabolism. Mink are able to both synthesize large amounts of glucose *de novo* and utilize high levels of dietary digestible carbohydrates, thereby tolerating large variations in dietary carbohydrate supply, but this function is limited (46). The GLU concentration increased with the increase in the ME levels in this study. Besides that, our data suggested that LDL, CHO, and TG contents were increased by ME levels, which revealed that mink tended to enhance liver lipid transportation to reduce liver damage by excessive lipid accumulation (10, 47). The plasma TP and Alb in the 35.97% CP and 18.18 MJ/kg ME diet were higher than those in the other diet groups and the concentrations of the serum GLU, TG, and LDL were higher than those in the other diet groups. Consequently, higher CP and ME levels in the diet can negatively affect protein and energy metabolism. The results of this study were consistent with the growth performance of the mink.

## Conclusion

Based on the above considerations, we concluded that the higher levels of CP and ME (35.97% and 18.18 MJ/kg) in the diet can promote body growth, increase nutrient digestibility, enhance N, energy intake and N retention, and promote the blood lipid metabolism in growing mink.

## Data Availability Statement

The raw data supporting the conclusions of this article will be made available by the authors, without undue reservation.

## Ethics Statement

All animals used in the study were treated following the guidelines established by the Council of China Animal Welfare. Protocols of the experiments were approved by the Animal Ethics Committee of the Chinese academy of agricultural sciences (CAAS). Written informed consent was obtained from the owners for the participation of their animals in this study.

## Author Contributions

FH wrote and WZ revised the article. JW collected the data. LC participated in the design of the study and contributed to the acquisition of data. All authors reviewed and approved the final manuscript.

## Funding

This study was supported by the National Innovation Program of Agricultural Science and Technology in CAAS (grant number CAAS-ASTIP-2019-ISAPS) and Start-up Fund for Scientific Research of Jilin Agricultural Science and Technology University (2021–7008). Gratitude is extended to the staff of the State Key Laboratory for Molecular Biology of Special Economic Animals and Animal Science and Technology College of Jilin Agricultural Science and Technology College for their assistance in carrying out these experiments.

## Conflict of Interest

The authors declare that the research was conducted in the absence of any commercial or financial relationships that could be construed as a potential conflict of interest.

## Publisher's Note

All claims expressed in this article are solely those of the authors and do not necessarily represent those of their affiliated organizations, or those of the publisher, the editors and the reviewers. Any product that may be evaluated in this article, or claim that may be made by its manufacturer, is not guaranteed or endorsed by the publisher.
